# Effects of different creep feed types on pre-weaning and post-weaning performance and gut development

**DOI:** 10.5713/ajas.17.0844

**Published:** 2018-10-23

**Authors:** Pil Seung Heo, Dong Hyuk Kim, Jae Cheol Jang, Jin Su Hong, Yoo Yong Kim

**Affiliations:** 1 School of Agricultural Biotechnology, and Research Institute of Agriculture and Life Sciences, Seoul National University, Seoul 08826, Korea

**Keywords:** Suckling Piglet, Weaning Pig, Creep Feed, Creep Feed Quality

## Abstract

**Objective:**

This experiment was carried out to determine the effects of different creep feed types on suckling performance and further adjustments to solid feed after weaning.

**Methods:**

A total of 24 multiparous sows and their litters were allotted to one of three treatment groups: i) provided highly digestible creep feed (Creep), ii) provided a pig weaning diet (Weaner), and iii) provided sow feed (Sow) as creep feed until weaning. After weaning, a total of 96 piglets were selected for evaluation of post-weaning performance.

**Results:**

For pre-weaning performance, the Creep treatment led to a significantly higher feed intake from 14 to 28 d (p<0.05) and higher body weight gain from 21 to 28 d than piglets that were provided other diets. However, after weaning, the Weaner treatment yielded a significantly higher feed intake and average daily gain than other treatments from 0 to 14 d after weaning (p<0.05); Creep treatment tended to generate lower villus heights in the duodenum than the other treatments (p = 0.07).

**Conclusion:**

Highly digestible creep feed improved pre-weaning performance, but feed familiarity and grain-based creep feed improved post-weaning performance.

## INTRODUCTION

Because of limited milk yield during late lactation and an expectation of easy adaptation to solid feed after weaning, providing creep feed has become common practice in modern pig management.

Creep feed is generally formulated with expensive ingredients such as milk by-products and plasma protein to help with nutrient uptake due to an undeveloped piglet digestive system [1], and various studies have used creep feed with highly digestible ingredients to maximize piglet growth until weaning [2–4]. However, when piglets are fed highly digestible creep feed, it is not clear whether weaning pigs can adjust to grain-based feed after weaning.

However, increasing familiarity to solid feed is one of the major purposes of creep feeding. To improve adaptation to solid feed after weaning, piglets need to consume cereal grains during the suckling period. Consumption of a grain diet induces maturation of digestive enzyme secretion [5,6], acid production [7], and nutrient absorption in the small intestine [5]. Moreover, piglets naturally start to consume sow feed from the third week after birth [8,9], meaning that grain-based creep feed can also be fed to young piglets during the late lactation period.

Consequently, this experiment was conducted to investigate the effects of different types of creep feed on the pre- and post-weaning performance of piglets by comparing three diets, namely, a highly digestible creep feed, a weaner diet, and a sow diet.

## MATERIALS AND METHODS

### Animal care

The protocol for this experiment was approved by the Ethical Committee for Institutional Animal Use and Care of the Seoul National University (SNU-16013-13). The experiment was conducted at the Jacob Swine Research Farm located in Eumseong-gun, North Chungcheong Province, Republic of Korea.

### Experimental animals and management

A total of 24 multiparous sows and their litters were used in this experiment. At 114 d of gestation, sows were allotted to a farrowing crate (2.5×1.8 m^2^) and a 4-hole stainless still feeder (60×20×25 cm) was installed in each farrowing crate for provision of creep feed. At 12 h postpartum, piglets were injected with Fe-dextran (150 ppm), the ears were notched, and needle teeth and tail were clipped. Boars were castrated at 3 d after birth. Experiments began at 14 d after birth, and each sow and their litters were assigned to one of three treatments according to body weight, back-fat thickness, and litter growth. To equalize the average litter growth of each treatment by day 14, cross-fostering was carried out before 24 h postpartum to standardize piglet numbers (11 piglets/L), average piglet weight (1.60±0.32 kg), and sex ratio. Each group was provided one of three different diets as creep feed. One group was provided creep feed that contained 61.8% milk product (Creep treatment), another group was given a pig weaner diet (Weaner treatment), and the third group was given sow feed (Sow treatment) through the creep feeder. At weaning, four male and four female piglets weighing close to the average body weight of each group were selected, and three males and three females (7.56±0.78 kg) were euthanized 4 d after weaning to analyze the condition of the gut. At the same time, a total of 96 piglets were selected to evaluate their adaptation to feed after weaning. Thirty-two piglets in each group were arranged in 8 piglets per pen according to body weight and sex. During the lactation period, sow and creep feed were provided *ad libitum*, and water was provided through nipples and water cups installed in sow crates and piglet areas.

The Creep treatment diet contained 61.8% milk product with 3,372 ME kcal/kg, 21% crude protein, 1.66% lysine, and 0.43% methionine. The Weaner treatment diet contained 12.5% milk product and 3,265 ME kcal/kg, 23.7% crude protein, 1.35% lysine, and 0.43% methionine. The Sow treatment, identical to the feed given to sows, contained 3,265 ME kcal/kg, 16.8% crude protein, 1.08% lysine, 0.28% methionine, 0.9% Ca, and 0.7% total P. To identify the creep feed eaters, 1% chromic oxide was mixed with each creep feed. From weaning until the second week after weaning, all piglets were fed the same weaner diet previously provided in the Weaner treatment. From the third to the fifth week after weaning, all piglets were fed a weaner II diet containing 3,265 ME kcal/kg, 20.9% crude protein, 1.15% lysine, and 0.3% methionine. Nutrients of the Sow and Weaner diets met or exceeded the NRC [10] nutrient requirements. The formulas and chemical composition of the creep diets used in this experiment are shown in Table 1.

### Sample collection and analysis

Sow and litter performance were recorded at 14, 21, and 28 d postpartum. The body weight, back-fat thickness, and feed intake of sows were measured, and the piglet weight and creep feed intake were recorded. The back-fat thickness of sows was measured at the P_2_ position using a digital Lean-Meter (Renco Co., Golden Valley, MN, USA). To identify the creep feed eaters and non-eaters, the fecal color was analyzed in the rectum with a cotton bud. The body weight and feed intake of the weaner pigs were recorded on the second and fifth weeks post-weaning. The individual body weight of each piglet was recorded, and individual feed intake was calculated from the feed intake of each pen according to Lindemann and Kim [11]. The intestinal morphology and microflora were analyzed 4 d after weaning. From the duodenum, mid-jejunum, and ileum, 20 mm specimens were excised and rinsed with physiological saline. Samples were then preserved in 10% neutral buffered formalin until analysis. Samples were dehydrated and cleaned with alcohol and xylene and then embedded in paraffin wax. Subsequently, 4 μm thick samples were sliced and stained with hematoxylin and eosin. Images of each sample were obtained using a Leica DM500 microscope (Leica microsystems, Solms, Germany) mounted with a Leica DFC 250 camera (Leica microsystems, Germany) to analyze villus height and crypt depth. To quantify the intestinal microflora, threshold cycles (Ct) of *Escherichia coli* (*E. coli*) *K88*+, *Lactobacillus casei*, *Lactobacillus plantarum*, and *Bacillus subtilis* in the ileum, cecum, colon, and rectum were observed using quantitative real-time polymerase chain reaction (qPCR). Digested samples from each digestive tract fraction were collected in a sterilized 50 mL conical tube and immediately frozen with liquid nitrogen. After freeze-drying, genomic DNA was extracted and isolated using bead-beating with a fecal DNA kit (UltraClean, MoBio, Carlsbad, CA, USA) and a total DNA extraction kit (G-spin, Intron Biotechnology, Seongnam, Korea). Microflora species-specific primers were designed and tested for specificity. The standards for the real-time qPCR were obtained by serial dilution of DNA plasmids from pure cultured transgenic *E. coli* containing the target DNA sequence. The qPCR was performed using a real-time PCR detection system (iCycler iQ, BioRad, Hercules, CA, USA).

### Statistical analysis

All collected data were analyzed using the mixed general linear model procedure of SAS (SAS Institute, Cary, NC, USA; 2004). To analyze sow and litter performance, sows and litters served as the experimental unit. Creep feed types were considered as a fixed effect, and parity and total born were considered a random effect. For data on the weaner period, each piglet served as an experimental unit, and their dams were considered a random effect. Significant differences were analyzed by least squares mean comparisons in the PDIFF option. Differences and suggestive differences between treatments were considered significant at p<0.05 and p<0.10, respectively.

## RESULTS

During the entire lactation period, the creep feed types did not affect sow performance, including body weight, back-fat thickness, feed intake, and weaning-to-estrus interval (Table 2).

The effects of creep feed types on suckling piglet performance and creep feed intake are presented in Table 3. Different types of creep feed did not alter the number of creep feed eaters, and practically all piglets were identified as eaters at 28 d regardless of creep feed type. From 14 to 21 days of age, litters under Creep treatment had the highest feed intake, and Creep treatment eaters tended to consume a higher level of creep feed than under Weaner and Sow treatments from 14 to 21 days of age (p = 0.10). From 21 to 28 days of age, the Creep treatment significantly (p<0.05) engendered a higher average daily gain than the other treatments.

Although the Creep treatment group showed the highest weaning weight, it also exhibited the lowest feed intake and weight gain after weaning (Table 4). The Weaner treatment group had a significantly higher feed intake than the other treatments from 0 to 14 d (p<0.05), and presented a significantly higher average daily weight gain than the Creep treatment (p<0.05). From 14 to 35 d, the Sow treatment group tended to show higher feed efficiency than the other treatments (p = 0.06).

The Creep treatment group tended to show a shorter duodenal villus height than the Weaner and Sow treatment groups (p<0.1) at 4 d after weaning (Table 5). The Sow treatment tended to promote a higher ileal crypt depth than the other treatments (p = 0.10). The real time PCR threshold cycle (Ct) values of microflora in the ileum and large intestine are presented in Table 6. The Sow group had the highest Ct value of *E. coli K88*+ in the ileum, colon, and rectum, although no significant differences were observed. The Sow group had significantly higher Ct values of *Lactobacillus plantarum* in the rectum than the Creep group (p<0.05). Similarly, the Sow group tended to have higher Ct values of *Bacillus subtilis* in the colon than the other groups (p<0.1).

## DISCUSSION

Creep feed consumption has a correlation with digestive maturity and poor milk yield [12], and increases linearly during the fourth week after birth [13]. In this study, piglets fed a highly digestible creep feed (Creep treatment) had a higher feed intake than piglets provided a weaner or sow diet as creep feed from 14 to 21 days of age, and their growth promoting effect was observed from 21 to 28 days of age. Similarly, Fraser et al [2] and Pajor et al [3] observed improved feed consumption from the third week after birth, but piglet growth did not improve during the period.

Piglets acquire most of their energy from consumption of maternal milk, and only a small amount from creep feed at the third or fourth week of age [1,14]. Whitelaw et al [15] revealed that creep feed consumption and growth of suckling piglets were not altered by dietary protein levels of between 14% and 22% until the third week. Additionally, Yan et al [16] also demonstrated that suckling piglet performance was not related to the energy density (4,000 vs 5,000 kcal/kg digestible energy) of creep feed until weaned at 21 d. Therefore, the digestible ingredient contents of creep feed are more important than the chemical composition in improving creep feed intake and growth performance of suckling piglets until weaning. In the present study, the weaner diet also contained 12.5% milk products, but pre-weaning feed intake did not differ from piglets fed the sow diet, indicating that more digestible contents are needed to accelerate creep feed consumption.

Providing creep feed could prevent sows from losing excessive body reserves during lactation by reducing piglet milk demand [17,18]. However, none of the observations showed differences among treatments in the current study. Differences of creep feed intake among treatments may not have been enough to affect sow performance. These results are in agreement with Sulabo et al [19], who also demonstrated the providing creep feed did not affect body weight or alter back-fat thickness.

Creep feed intake is strongly correlated to post-weaning feed intake and weight gain [12,20]. Pre-weaning creep feed intake stimulates further post-weaning feed intake and decreases the time-to-consumption of post-weaning diets [7]. However, in the current study, pigs were provided a weaner diet until weaning had a significantly higher feed intake than the other pigs during the first two weeks after weaning. Moreover, pigs fed highly digestible creep feed during the suckling period had the lowest weight gain and feed intake from 0 to 14 d after weaning despite having the heaviest weaning weight with the highest pre-weaning feed intake. This result indicated that feed familiarity is more important than pre-weaning creep feed intake when the diet changed at weaning. Yan et al [16] suggested that effects of creep feeding are considerably clearer at post-weaning than pre-weaning because of oral tolerance to solid feed.

Pigs generally undergo drastic changes in the gastrointestinal tract after weaning. Their intestinal villus thickens and shortens, and the crypt depth deepens when adjusting to solid feed. Several studies have shown that solid feed stimulates acid production and digestive enzyme activity [6,7]. Therefore, providing creep feed during the suckling period helps the development of amylase and protease activity in the gut, and provides an advantage for the utilization of solid feed and prevention of denial of feed consumption after weaning [5].

Van Beers-Schreurs et al [21] reported that villus atrophy after weaning was caused by solid feed and amount of feed intake. In the current study, a decrease in the duodenal villus height was observed in pigs fed a highly digestible creep feed during the suckling period, and pigs provided sow feed as creep feed tended to have the deepest crypt depth in the ileum. These results are also in agreement with Pluske et al [22] who showed that pigs fed a starter diet after weaning had reduced villus height and increased crypt depth 5 d after weaning, while pigs fed ewe milk maintained villus height and crypt depth. Therefore, the results of small intestinal morphology support the observations of post-weaning performance in the current study.

Jensen [23] reported an increase in coliform bacteria and a decrease in *Lactobacilli* in the rectum 4 d after weaning, and Franklin et al [24] showed a similar result in the distal intestine 3 d after weaning. In the current study, the Sow treatment group presented the lowest population of *Lactobacillus plantarum* in the rectum and tended to show lower *Bacillus subtilis* in the colon. However, no significant difference was observed in coliform bacteria among treatments, with the Sow treatment group additionally showing a reduction in the ileum, colon, and rectum. Rantzer et al [25] reported reduced levels of fecal hemolytic *E. coli* with feed restriction after weaning. Although the Sow treatment group showed feed intake similar to the Creep treatment group during the 2 weeks after weaning in the current study, Sow treatment may have resulted in lower feed intake shortly after weaning.

In conclusion, highly digestible creep feed may improve pre-weaning piglet feed consumption and growth, but feed familiarity and rough grain-based feed benefits post-weaning performance when diet changes with weaning.

## Figures and Tables

**Table 1:** Formula and chemical composition of creep diets in the experiment

Ingredients	Creep (%)	Weaner (%)	Ingredients	Sow (%)
	
Suprex corn	16.90	20.88	Corn	79.61
Soybean meal	3.10	30.96	Soybean meal	14.99
Fermented soybean meal	6.70	8.36	Sugar molasses	0.50
Whey powder	28.88	3.50	Soy oil	0.51
Whey protein concentrate	13.24	-	L-lysine-HCl	0.24
Cheese powder	6.78	-	DL-methionine	0.04
Fish meal	4.80	-	Dicalcium phosphate	2.49
Lactose	12.90	9.00	Limestone	0.85
Barley	3.39	24.11	Vitamin premix1)	0.20
Soy oil	1.00	0.43	Mineral premix2)	0.10
Monocalcium phosphate	0.22	0.97	Salt	0.32
Limestone	0.79	0.98	Choline Cl (50%)	0.15
L-lysine-HCl	0.33	0.14		
DL-methionine	0.07	0.03		
Vitamin premix3)	0.25	0.12		
Mineral premix4)	0.25	0.12		
Salt	0.20	0.20		
Choline-Cl (25%)	0.10	0.10		
ZnO	0.10	0.10		
Sum	100.00	100.00		100.00
Chemical composition5)				
Metabolizable energy (Kcal/kg)	3,372.08	3,265.01		3,265.00
Crude protein (%)	21.00	23.70		12.90
Lysine (%)	1.66	1.35		0.74
Methionine (%)	0.43	0.35		0.23
Calcium (%)	0.90	0.80		0.90
Total phosphate (%)	0.70	0.65		0.70

1) Supplied the following per kilogram of complete feed: 10,000 IU of vitamin A; 1,500 IU of vitamin D3; 35 IU of vitamin E; 3 mg of vitamin K; 4 mg of vitamin B_2_; 3 mg of vitamin B_6_; 15 μg of vitamin B_12_; 10 mg of pantothenic acid; 50 μg of biotin; 20 mg of niacin; 500 μg of folic acid.

2) Supplied the following per kilogram of complete feed: 75 mg of Fe; 20 mg of Mn; 30 mg of Zn; 55 mg of Cu; 100 μg of Se; 250 mg of Co; 250 mg of I.

3) Supplied the following per kilogram of complete diet: 8,000 IU of vitamin A; 1,600 IU of vitamin D_3_; 32 IU of vitamin E; 2.4 mg of vitamin K; 3.2 mg of vitamin B_2_; 12 μg of vitamin B_12_; 8 mg of pantothenic acid; 64 μg of biotin; 16 mg of niacin.

4) Supplied the following per kilogram of complete diet: 127.3 mg of Fe; 54.1 mg of Cu; 24.8 mg of Mn; 84.7 mg of Zn; 0.3 mg of Co; 0.3 mg of I; 0.1 mg of Se.

5) Calculated value.

**Table 2:** Effect of different creep feed types on sow performance

Items	Treatment			SEM	p-value

	Creep	Weaner	Sow		
Body weight (kg)					
Initial (day 14)	235.7	235.1	235.8	3.88	0.88
Day 21	233.8	234.1	233.8	4.16	0.96
Day 28	231.1	229.4	230.8	3.97	0.85
Body weight change	−4.6	−5.8	−5.0	0.91	0.96
Back-fat thickness (mm)					
Initial (day 14)	21.4	22.4	22.4	1.05	0.91
Day 21	20.7	22.1	21.2	1.00	0.85
Day 28	20.1	21.4	20.3	0.98	0.85
Back-fat change	−1.3	−1.0	−2.1	0.27	0.48
Average daily feed intake (kg)					
Day 14 to 21	5.55	5.71	6.03	0.22	0.46
Day 21 to 28	6.34	5.88	6.67	0.19	0.26
Weaning-to-estrus interval (d)	5.19	4.86	4.81	0.13	0.44

SEM, standard error of the mean.

**Table 3:** Effect of different creep feed types on piglet growth and creep feed intake

Items	Treatment			SEM	p-value

	Creep	Weaner	Sow		
Average body weight (kg)					
Initial (Day 14)	3.88	3.84	3.87	0.053	0.97
Day 21	5.43	5.26	5.24	0.097	0.81
Day 28	7.43	6.83	7.00	0.134	0.41
Average daily gain (g)					
Day 14 to 21	221	202	196	8.2	0.47
Day 21 to 28	286^A^	225^B^	251^B^	9.8	0.01
Average daily creep feed intake of litters (g/d)					
Day 14 to 21	197.7	143.2	94.2	19.99	0.12
Day 21 to 28	684.6	642.3	533.3	63.65	0.58
No. of piglets consuming creep feed (piglets/L)					
Day 21	5.5	6.8	5.3	0.63	0.62
Day 28	10.3	10.0	10.3	0.32	0.92
Average daily creep feed intake of eaters1) (g/d)					
Day 14 to 21	36.1^a^	22.9^b^	23.5^b^	3.01	0.10
Day 21 to 28	65.8	61.7	50.9	5.81	0.54

SEM, standard error of the mean.

1) Average value of feed intake/No. of Eaters of each litter.

A,B Means in the same row with different superscript letters were significantly different (p<0.05).

a,b Means in the same row with different superscript letters were significantly different (p<0.10).

**Table 4:** Effect of supplementation of different creep feed types during lactating period on piglet performance after weaning

Items	Treatment			SEM	p-value

	Creep	Weaner	Sow		
Body weight (kg)					
At weaning	7.87	7.38	7.53	0.170	0.48
Day 14	10.91	11.04	10.76	0.210	0.87
Day 35	19.74	19.94	20.19	0.302	0.83
Average daily weight gain (kg)					
Day 0 to 14	217^B^	261^A^	231^AB^	7.0	0.03
Day 14 to 35	421	424	449	7.5	0.23
Overall	339	359	362	6.0	0.24
Average daily feed intake (kg)					
Day 0 to 14	328^B^	369^A^	333^B^	6.7	0.02
Day 14 to 35	784	799	814	12.0	0.60
Overall	602	627	621	8.8	0.46
Gain/feed ratio					
Day 0 to 14	0.65	0.70	0.67	0.010	0.23
Day 14 to 35	0.53^b^	0.53^b^	0.55^a^	0.003	0.06
Overall	0.56	0.57	0.58	0.003	0.14

SEM, standard error of the mean.

A,B Means in the same row with different superscript letters were significantly different (p<0.05).

a,b Means in the same row with different superscript letters were significantly different (p<0.10).

**Table 5:** Effect of supplementation of different creep feed types during the lactating period on the morphology of the small intestine 4 d after weaning

Items	Treatment			SEM	p-value

	Creep	Weaner	Sow		
Duodenum					
Villus height (μm)	572^b^	738^a^	733^a^	35.7	0.07
Crypt depth (μm)	455	481	536	21.5	0.31
Villus/crypt ratio	1.26	1.54	1.39	0.056	0.11
Jejunu					
Villus height (μm)	518	574	670	33.0	0.17
Crypt depth (μm)	374	410	449	17.8	0.24
Villus/crypt ratio	1.39	1.41	1.50	0.052	0.68
Ileum					
Villus height (μm)	464	463	566	27.2	0.24
Crypt depth (μm)	332^b^	318^b^	390^a^	14.2	0.10
Villus/crypt ratio	1.39	1.46	1.45	0.041	0.85

SEM, standard error of the mean.

A,B Means in the same row with different superscript letters were significantly different (p<0.05).

a,b Means in the same row with different superscript letters were significantly different (p<0.10).

**Table 6:** Effect of different creep feed types during the lactating period on the microflora population1) in the ileum and large intestine 4 d after weaning

Items	Treatment			SEM	p-value

	Creep	Weaner	Sow		
*Escherichia coli K88*+					
Ileum	20.85	21.04	32.01	3.006	0.17
Cecum	25.75	26.32	24.48	2.548	0.98
Colon	27.46	21.14	29.36	2.510	0.43
Rectum	20.94	27.80	30.86	2.404	0.22
*Lactobacillus casei*					
Ileum	15.95	18.02	17.26	1.100	0.77
Cecum	16.07	16.37	18.03	0.677	0.30
Colon	17.05	17.35	18.73	0.432	0.25
Rectum	17.16	17.30	18.02	0.616	0.85
*Lactobacillus plantarum*					
Ileum	16.54	16.02	14.84	0.600	0.37
Cecum	14.82	15.70	15.09	0.510	0.75
Colon	15.14	15.19	16.52	0.560	0.54
Rectum	13.53^B^	14.35^AB^	16.76^A^	0.574	0.04
*Bacillus subtilis*					
Ileum	26.70	23.03	32.38	1.812	0.31
Cecum	25.18	26.52	28.01	1.421	0.78
Colon	24.75^b^	25.17^b^	31.89^a^	1.384	0.07
Rectum	26.69	24.85	30.26	1.104	0.50

SEM, standard error of the mean.

1) Microflora populations are represented with the Ct value from the real time polymerase chain reaction.

AB Means in the same row with different superscript letters were significantly different (p<0.05).

ab Means in the same row with different superscript letters were significantly different (p<0.10).
